# Prevalence of Selected Eye Diseases Using Data Harvested from Ophthalmic Checkup Examination of a Cohort of Two Thousand Middle Eastern and North African Subjects

**DOI:** 10.1155/2018/8049475

**Published:** 2018-03-04

**Authors:** Nehal M. Samy El Gendy, Ahmed A. Abdel-Kader

**Affiliations:** Ophthalmology Department, Kasr Al Ainy Medical School, Cairo University, Cairo, Egypt

## Abstract

**Purpose:**

To highlight the prevalence of selected ophthalmic diseases accidentally discovered at first-time screening of a large sample of patients from the Middle East and North Africa visiting a large referral university hospital checkup unit based in Cairo.

**Material and Methods:**

A cross-sectional study of two thousand and thirteen subjects coming for routine ophthalmic medical checkups from different Middle East countries (mainly Egypt, Sudan, and Yemen). Patients were evaluated for prevalence of diabetic retinopathy, glaucoma, ocular hypertension, cataract, and amblyopia. Patients' demographic data and medical history were collected. Complete ophthalmic examination was performed. Investigations were done when needed to confirm suspected conditions.

**Results:**

The study included 1149 males and 864 females. 652 Sudanese patients, 568 Yemeni patients, 713 Egyptian patients, and 63 patients from different Gulf and North African countries like Saudi Arabia, Qatar, Libya, and Jordan. Sudanese patients showed a higher percentage of glaucoma (13.3%) and ocular hypertension (8.3%). Yemeni patients showed the highest prevalence of amblyopia (6.7%), diabetic retinopathy (8.6%), and cataract (4.2%). The group of relatively higher economic classification seemed to show fewer prevalences of these ophthalmic conditions. Yemeni patients tended to have a high percentage of persistent myelinated nerve fibers.

**Conclusion:**

Different ophthalmic conditions were discovered for the first time at the general checkup clinic. Certain conditions were more common than others in certain countries. The lack of regular checkups and the unavailability of medical services due to low to moderate socioeconomic status as well as political turbulence may account for the delay in initial diagnosis of many treatable conditions.

## 1. Introduction

Economic deprivation in developing countries and/or countries with recent political instability as in the Middle East region has led to the development of pockets of poverty and extreme health neglect [1]. With the recent political changes in the Middle East and North Africa (MENA) that affected multiple Arab countries, proper healthcare including eye care has become difficult to access by all socioeconomic standards. With few exceptions like Qatar, Jordan, United Arab Emirates, and Saudi Arabia, most Arab countries in the Middle East and North Africa are considered low- to lower middle-economy countries according to the World Bank classification [2].

Racial and cultural factors also affect prevalence of diseases among different populations. However, little has always been known about disease patterns in the Middle East and North Africa patients. Of the few studies that were done in the MENA region was a study done in Al-Madinah Al-Munawarah in Saudi Arabia where out of 690 diabetic patients, 36.1% were found to have diabetic retinopathy of which 6.4% had proliferative disease [3]. Another cross-sectional study was done in United Arab Emirates where diabetic retinopathy was discovered to be present in 19% of 513 diabetic patients in Al Ain city. Of these patients, 74% were totally unaware of their retinal condition [4].

In Sudan, a recent study alarmed that the rate of type 2 diabetes mellitus (DM) was increasing among children and young adolescents especially obese ones of families of high socioeconomic status. Out of the 985 patients included in that study, 38 (4%) were labeled as having type 2 DM, of which 35 (92.1%) had the onset of disease between 11 and 18 years of age [5]. The study, however, did not mention the retinal condition of these children. In Yemen, a study done on 350 adults with DM showed the prevalence of diabetic retinopathy (DR) to be 55%, of which 17% had proliferative retinopathy [6]. In Egypt, at a hospital-based study, the prevalence of DR among diabetic patients above 18 years of age was 20.5%. In this cross-sectional survey study, 82% of patients were unaware of the hazards of diabetes on the eyes [7].

Very little is yet written about the prevalence of glaucoma and ocular hypertension in Egypt, Sudan, Yemen, and other countries. Sudan, Yemen, and Egypt are developing countries in which medical awareness of diseases that could insidiously affect the eyes and lead to substantial visual loss is deficient.

New Kasr Al Ainy hospital checkup clinic receives patients from all over the Middle East countries. However, majority comes from Egypt, Sudan, and Yemen. The hospital also receives fewer patients from Saudi Arabia, Qatar, Libya, and Jordan. Patients who presented to this clinic may have been known to have diabetes or hypertension, but all were supposed to be ophthalmologically free. It was the scarcity of literature about screening for eye disease in the MENA region that compelled the authors to conduct this study. The study aimed at pointing out, among this large number of patients coming from different MENA countries, the prevalence of selected ophthalmic diseases that are missed and/or accidentally discovered at first checkup.

## 2. Materials and Methods

Our cross-sectional study included a total of 2013 patients. All patients presented for the first time to the same physician in the weekly checkup ophthalmology clinic at Kasr Al Ainy New Teaching Hospital of Cairo University. The study included all patients who attended this checkup clinic from January 2014 to December 2016. Cairo University Hospital's ethics committee approved the study. The study adhered to the tenets of the Declaration of Helsinki [8].

Patients with a known eye disease, previous eye surgery, or interventional treatment like laser treatment or anti-VEGF injection were excluded from the study. Patients' demographic data as nationality, age, sex, and social status were documented. Medical history including diabetes mellitus, hypertension, and other systemic diseases was documented. Ocular examination included corrected distant visual acuity (CDVA), intraocular pressure (measured by air puff and confirmed by Goldman applanation tonometer), slit-lamp biomicroscopy and dilated fundus examination using indirect ophthalmoscope, and slit-lamp biomicroscopy with 90 D lens.

Investigations were done when needed to confirm suspected conditions. Field of vision, corneal pachymetry, and retinal nerve fiber layer analysis by optical coherence tomography were done in suspected glaucoma patients and to diagnose ocular hypertension.

Ocular hypertension was defined as IOP measured by Goldmann applanation tonometer higher than 22 mmHg after correction for central corneal thickness (CCT) using Kohlhaas et al.'s correction formula with Dresdner correction table [9], with normal visual field, normal appearance of ONH, and normal retinal nerve fiber layer thickness (RNFL) by OCT [10].

Glaucoma was diagnosed in our study based on the International Society of Geographical and Epidemiological Ophthalmology (ISGEO) definitions for glaucoma: definition 1, optic nerve head structural damage (vertical cup/disc ratio “VCDR” ≥ 97.5th percentile ≥ 0.7, asymmetry cup/disc ratio “CDR” ≥ 0.2, confirmed by OCT with vertical RNFL defect) and glaucomatous visual field (three contiguous points more than 5 dB depressed or one point more than 10 dB depressed and either corrected pattern standard deviation (CPSD) in the 30-2 full-threshold test or PSD in the SITA-standard C-30 test with a *P* value index < 0.05 or glaucoma hemifield test (GHT) index outside normal limits); definition 2, advanced optic nerve damage (VCDR ≥ 99.5th percentile ≥ 0.8, confirmed by OCT) if VF could not be performed; and definition 3, if vision < 0.05 with IOP ≥ 99.5th percentile = 26 mmHg, VF could not be performed and optic disc could not be seen [11]. Normative data for IOP and VCDR was based on data from the Egyptian Society for the Glaucomas and other glaucoma prevalence surveys on African and Arab populations [12–15].

Fundus fluorescein angiography and optical coherence tomography were done when needed for diabetic patients with retinopathy and/or maculopathy. Testing for amblyopia included best-corrected visual acuity (BCVA) and pinhole testing. Amblyopia was diagnosed unilaterally if BCVA < 0.63 decimal with a difference of 2 or more lines between the two eyes in the absence of any ocular organic disease or bilaterally if BCVA < 0.63 in the presence of high ametropia (hyperopia > 4 D, astigmatism > 2 D, or myopia > 6 D) in the absence of any ophthalmological abnormality [16].

Cataract was diagnosed with slit-lamp examination and classified based on lens opacity classification system II (LOCS II) for being simpler, reproducible, and more suitable for prevalence studies [17, 18]. Visually significant cataract was defined by any LOCS II grading ≥ 2, BCVA < 0.5 (Snellen's decimal notation), and cataract as the primary cause of vision impairment.

Statistical data were described in frequency and percentages for categorical data and mean ± SD for numerical data using SPSS version 24 (SPSS Inc., Chicago, IL, USA).

## 3. Results

### 3.1. Demographic Data and Medical History

Our study sample included patients from Sudan, Yemen, Egypt, Qatar, Saudi Arabia, Libya, and Jordan. 652 patients were Sudanese (of which 57.1% were males and 42.9% were females), 567 were Yemeni (of which 69.5% were males and 30.5% females), and 731 were Egyptian (of which 48.1% were males and 51.9% females). Sixty-three patients held other nationalities (Qatari, Saudi Arabian, Libyan, and Jordanian) and were put together in one group due to the small number of patients (of which 49.2% were males and 50.8% females).

The mean age (in years) of patients coming for routine checkup was 51.8 ± 11.1 years among Sudanese patients, 52.5 ± 12 years among Yemeni patients, 57.1 ± 12.5 years among Egyptian patients, and 52.3 ± 13.7 years among the rest of the nationalities.

Among Egyptian patients, 9.2% gave a history of diabetes, and 12.2% of hypertension. Among Sudanese patients, 13.2% gave a history of diabetes, and 23.6% of hypertension. Among Yemeni patients, 9.9% gave a history of diabetes, and 6.2% of hypertension. Patients having other nationalities (Saudi Arabian, Libyan, Jordanian, and Qatari) comprised a small percentage of our sample (3.1%). 4.7% of them gave a history of diabetes and 3.2% gave a history of hypertension.  Table 1 summarizes demographic data and medical history of patients.

### 3.2. Glaucoma and Ocular Hypertension

A total of 13.3% were discovered to be glaucomatous for the first time and were confirmed by further tests. It is interesting knowing that 48.3% of glaucomatous patients were below 45 years of age and 26.4% were below 40 years of age. 8.3% were discovered to have ocular hypertension.

1.9% of Egyptian patients were discovered to be glaucomatous for the first time that was confirmed by further tests as perimetry, OCT, and central corneal thickness. 1.6% of patients were discovered to have ocular hypertension. 4.1% of Yemeni patients were discovered to be glaucomatous for the first time and were confirmed by further tests. 2.5% were discovered to have ocular hypertension. Sudanese patients showed the highest percentage of glaucoma (13.3%) and ocular hypertension (8.3%). Patients having other nationalities (Saudi Arabian, Libyan, Jordanian, and Qatari) showed 3.2% ocular hypertension with no prevalence of glaucoma.

### 3.3. Diabetic Retinopathy (DR)

5.7% of Egyptian patients were found to have different stages of diabetic retinopathy. These represented 62.7% of diabetic patients. 3.5% of Sudanese patients were found to have different stages of diabetic retinopathy. These represented 26.7% of diabetic patients. 8.6% of Yemeni patients were found to have different stages of diabetic retinopathy. These represented 87.5% of diabetic patients.  Table 2 summarizes the prevalence of different stages of diabetic retinopathy according to the international clinical diabetic retinopathy scale [19]. Grading was based on findings on the worst eye.

### 3.4. Amblyopia

Yemeni patients showed the highest prevalence of amblyopia being 6.7% compared to 1.6% in Egyptian patients and 1.2% in Sudanese patients. The etiology in all cases was error of refraction.

### 3.5. Cataract

Yemeni patients showed the highest prevalence of visually significant cataract being 4.2% compared to 1.4% in Egyptian patients and 1.8% in Sudanese patients.

Patients of other nationalities (Saudi Arabian, Libyan, Jordanian, and Qatari) comprised a small percentage of our sample. None of them was diagnosed with glaucoma, yet 3.2% of them had ocular hypertension. None of the patients in this group had evidence of diabetic retinopathy, amblyopia, or significant cataract.

As opposed to all other nationalities, 9.9% of Yemeni patients were found to have persistent myelinated nerve fibers.

**Figure 1:**
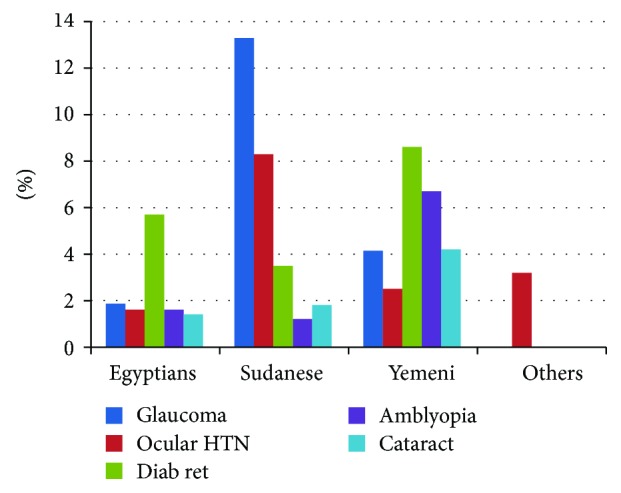
Bar chart comparing the percentage (*y*-axis) of ocular conditions met within all four groups (*x*-axis).

Table 3 summarizes prevalence of different ocular conditions among four groups.  Figure 1 shows a bar graph comparing the percentages of ocular findings between the four groups.

### 3.6. Normal Subjects

Our study evaluated the prevalence of selected eye diseases with global health concern. Several other eye diseases were not documented in our study. Patients with ocular hypertension were not considered normal as they need closer follow-up or antiglaucoma medications. Subjects checked as normal were 463 (71%) in Sudanese patients, 410 (72.3%) in Yemeni, 637 (87.1%) in Egyptians, and 60 (95.2%) in the minor nationality group, considering cases with ocular hypertension as abnormal, as it may need further follow-up.

## 4. Discussion

Different ophthalmic diseases can be discovered through efficient screening. Developing countries or countries with low to medium income usually lack the luxury of effective screening programs. Kasr Al Ainy Teaching Hospital, Cairo, Egypt, has always been a favored referral center for its renowned “checkup program” which was sought by citizens from all the MENA countries. It is an economic comprehensive program served by numerous consultants and includes screening of all systems of the body, including the special senses. The checkup ophthalmology clinic is well equipped with basic ophthalmic examination instruments. Most of its patients are unaware of their eye diseases. Patients attend the checkup program aiming for prevention and early detection of diseases benefitting from economic value and professional services. The authors thought about the opportunity to compare data from different MENA countries regarding prevalences of selected eye diseases.

One of the limitations of our study is being held on patients from the general checkup clinic at a general hospital. Patients with systemic illnesses and diseases tend more to seek checkup clinics. Our study is not a screening study on a naive population. However, bias was limited by excluding patients with known ophthalmic disease or illness from our statistical analysis.

A higher percentage of glaucoma and ocular hypertension was found in Sudanese patients. The percentage was almost triple that of Yemeni patients and six times that of Egyptian patients. Having a dark skin color, Sudanese are definitely at a higher risk of primary open-angle glaucoma. This ratio almost agrees with that of open-angle glaucoma in African Americans compared to non-Hispanic whites, which is almost fourfold [20]. Early onset glaucoma is a thing that should raise caution in dealing with Sudanese patients especially during screening programs. Only 16.9% of the patients had the knowledge that glaucoma should be screened after the age of 40 years. In our study, we recommend early screening of North African patients, by the age of 35 years.

On the other hand, Sudanese patients had the lowest percentage of diabetic retinopathy, compared to Egyptian and Yemeni patients. This may partially be explained by the relatively active lifestyle of most Sudanese citizens (who work in agriculture and/or trading of goods) rather than bureau work or long desk hours that may be present in other MENA countries. It may also be attributed to the relatively low percentage of obesity in Sudan (13.8% in males and 26% in females in 2017) [21] when compared to Egypt and the gulf area (ranges from 74% to 86% in women and 69% to 77% in men) where 70% or more of adults above 15 years are either obese or overweight according to the WHO reports in 2015 [22].

The fact that Yemeni patients have a higher percentage of amblyopia and significant cataract may be attributed to the delay in seeking medical advice and the unavailability of medical services in most areas of Yemen. Yemen is also largely a tribal community where a lot of cousin and family marriages take place, a point that may contribute through genetic factors to the occurrence and passing on of certain eye conditions including large refractive errors and myelinated nerve fibers [23]. However, to prove or negate this point needs further detailed study.

Selected ophthalmic conditions were not detected in significant percentages in subjects from Jordan, Qatar, Saudi Arabia, and UAE. The authors attributed this to the relatively high economic status of these countries and the availability of medical services (including medical insurance and good national health system) in these countries which in turn did not drive citizens to seek medical advice elsewhere.

Different ophthalmic conditions were discovered for the first time at the general checkup clinic. Certain conditions were more common than others in certain countries. The lack of regular checkups and the unavailability of medical services associated with low to moderate socioeconomic status as well as political turbulence may account for the delay in initial diagnosis of many treatable conditions. Sudanese patients are obviously at a higher risk for glaucoma and ocular hypertension than other countries in the Middle East. The authors thus recommend that a glaucoma screening program be implemented for the detection of ocular hypertension, glaucoma suspects, and open-angle glaucoma in Sudan.

Yemeni patients have a higher percentage of amblyopia, a sequel of untimely diagnosis, or missed diagnosis of refractive errors. This is a largely preventable condition if routine eye screening for refractive errors is adopted before school entry or screening campaigns are set up to screen kids at different elementary school grades. Persistent myelinated nerve fibers were also a common finding in patients from Yemen, a finding that may have a genetic predisposition and is a point that needs further investigation.

## Figures and Tables

**Table 1:** Demographic data and medical history of patients.

Total number = 2013	Egypt	Sudan	Yemen	Others
Number (%)	731 (36.3%)	652 (32.4%)	567 (28.2%)	63 (3.1%)
Male	352 (48.1%)	372 (57.1%)	394 (69.5%)	31 (49.2%)
Female	379 (51.9%)	280 (42.9%)	173 (30.5%)	32 (50.8%)
Age, years (mean ± SD)	57.1 ± 12.5	51.8 ± 11.1	52.5 ± 12	52.3 ± 13.7
Diabetes	67 (9.2%)	86 (13.2%)	56 (9.9%)	3 (4.7%)
Hypertension	89 (12.2%)	154 (23.6%)	35 (6.2%)	2 (3.2%)

**Table 2:** Prevalence of different stages of diabetic retinopathy (DR) in diabetic patients and mean duration of diabetes.

	Egypt (number = 67)	Sudan (number = 86)	Yemen (number = 56)
Number of DR	25 (37.3%)	63 (73.3%)	7 (12.5%)
Duration of DM (years)	9.4 ± 6.6	10.2 ± 7.3	9.1 ± 4.8
NPDR	30 (44.8%)	15 (17.4%)	32 (57.1%)
Duration of DM (years)	14.7 ± 7.8	15.9 ± 5.9	14.5 ± 7.1
PDR	8 (11.9%)	5 (5.8%)	11 (19.6%)
Duration of DM (years)	19.2 ± 3.3	18.6 ± 2.5	18.1 ± 3.4
Advanced DR	4 (6%)	3 (3.5%)	6 (10.7%)
Duration of DM (years)	25.3 ± 3.5	22.5 ± 2.5	23.5 ± 4.9

**Table 3:** Prevalence of selected diseases in four groups.

Total number = 2013	Egypt (number = 731)	Sudan (number = 652)	Yemen (number = 567)	Others (number = 63)
Glaucoma	14 (1.9%)	87 (13.3%)	23 (4.1%)	0
Ocular hypertension	12 (1.6%)	54 (8.3%)	14 (2.5%)	2 (3.2%)
Diabetic retinopathy (% total)	42 (5.7%)	23 (3.5%)	49 (8.6%)	0
Diabetic retinopathy (% diabetics)	62.7%	26.7%	87.5%	0
Amblyopia	12 (1.6%)	8 (1.2%)	38 (6.7%)	0
Cataract	10 (1.4%)	12 (1.8%)	24 (4.2%)	0
