# Erratum to “Prevalence of Selected Eye Diseases Using Data Harvested from Ophthalmic Checkup Examination of a Cohort of Two Thousand Middle Eastern and North African Subjects”

**DOI:** 10.1155/2018/6168597

**Published:** 2018-07-22

**Authors:** Nehal M. Samy El Gendy, Ahmed A. Abdel-Kader

**Affiliations:** Ophthalmology Department, Kasr Al Ainy Medical School, Cairo University, Cairo, Egypt

In the article titled “Prevalence of Selected Eye Diseases Using Data Harvested from Ophthalmic Checkup Examination of a Cohort of Two Thousand Middle Eastern and North African Subjects” [1], there was an error in the first row of Table 2, where “Number of DR” should be corrected to “No DR.” This occurred due to a production error. The corrected table is shown below.

## Figures and Tables

**Table 2:** Prevalence of different stages of diabetic retinopathy (DR) in diabetic patients and mean duration of diabetes.

	Egypt (number = 67)	Sudan (number = 86)	Yemen (number = 56)
No DR	25 (37.3%)	63 (73.3%)	7 (12.5%)
Duration of DM (years)	9.4 ± 6.6	10.2 ± 7.3	9.1 ± 4.8
NPDR	30 (44.8%)	15 (17.4%)	32 (57.1%)
Duration of DM (years)	14.7 ± 7.8	15.9 ± 5.9	14.5 ± 7.1
PDR	8 (11.9%)	5 (5.8%)	11 (19.6%)
Duration of DM (years)	19.2 ± 3.3	18.6 ± 2.5	18.1 ± 3.4
Advanced DR	4 (6%)	3 (3.5%)	6 (10.7%)
Duration of DM (years)	25.3 ± 3.5	22.5 ± 2.5	23.5 ± 4.9
